# Evaluation of Immunophenotypic Characteristics and Quantitative Differences of Telocytes Between Invasive Breast Cancer Subtypes

**DOI:** 10.3390/life15071040

**Published:** 2025-06-30

**Authors:** Nilgün Öksel, İbrahim Halil Erdoğdu, Ömer Faruk Akgül, Merve Bulut, Özlem Yersal

**Affiliations:** 1 Department of Histology and Embryology, Faculty of Medicine, Gaziosmanpaşa University, Tokat 60030, Turkey; nilgun.yersal@gop.edu.tr (N.Ö.); mervemalatya971@gmail.com (M.B.); 2 Department of Pathology, Faculty of Medicine, Adnan Menderes University, Aydın 09100, Turkey; ibrahimhalilerdogdu@gmail.com; 3 Department of Medical Oncology, Atatürk Public Hospital, Aydın 09020, Turkey; dromerfarukakgul@gmail.com; 4 Department of Medical Oncology, Ümit Hospital, Eskişehir 26180, Turkey

**Keywords:** breast cancer, telocyte

## Abstract

Background: Breast cancer is the most commonly diagnosed neoplasm in women and is classified into different molecular subtypes based on the expression characteristics of estrogen and progesterone receptors (ERs and PRs) and human epidermal growth factor 2 (HER2, ERBB2): Luminal A, Luminal B, HER2(+), and triple-negative breast cancer (TNBC). Telocytes, a new type of stromal cell, provide structural support for the preservation of organ integrity and play a crucial role in the tumor microenvironment. In this study, we evaluated telocyte counts and expression profiles among breast cancer subtypes. Methods: The quantitative differences between telocytes in three subtypes of invasive breast cancer were assessed via immunohistochemistry, using vimentin, CD10, CD34, and c-Kit antibodies. Results: Vimentin(+), CD10(+), CD34(+), and c-Kit(+) telocyte counts were significantly higher in the Luminal and HER2(+) groups than in TNBC (*p* = 0.000 for vimentin, CD10, CD34, and c-Kit in Luminal vs. TNBC; *p* = 0.006 for CD34 in HER2(+) vs. TNBC). CD10(+), CD34(+), and c-Kit(+) telocyte counts were significantly higher in ER(+) than in ER(–) patients (*p* = 0.006, *p* = 0.000, and *p* = 0.009, respectively) and in PR(+) than in PR(–) patients (*p* = 0.018, *p* = 0.000, and *p* = 0.044, respectively). The presence of ER/c-Kit(+) telocytes was demonstrated, and c-Kit(+) telocyte counts were significantly lower in tumors larger than 5 cm than in those measuring 2–5 cm (*p* = 0.032). Conclusions: Our results showed quantitative differences and marker expression profiles for telocytes between different breast cancer molecular subtypes. c-Kit(+) telocytes may contribute to the regulation of tumor size.

## 1. Introduction

Breast cancer is the most frequent cancer diagnosed among women. Despite improvements in early screening methods and advances in adjuvant therapy, breast cancer is one of the main causes of cancer deaths due to the development of metastases [1]. Breast cancer is classified into different molecular subtypes based on the expression characteristics of estrogen and progesterone receptors (ER and PR) and human epidermal growth factor receptor 2 (HER2; ERBB2): Luminal A, Luminal B, HER2(+), and triple-negative breast cancer (TNBC) [2].

In recent years, telocytes, a novel type of stromal cell, have received attention due to their potential structural and regulatory functions in several normal and pathological tissues, including breast cancer [3]. Telocytes can be identified morphologically by small, piriform, spindle- or triangular-shaped cell bodies and long cytoplasmic extensions called telopodes. They do not have a widely accepted molecular marker profile; their expression may vary across different organs [4]. Telocytes are associated with fibroblasts, muscle cells, immune cells, nerve endings, and blood vessels in many tissues through hetero-cellular connections. To date, their presence in many organs has been demonstrated, their phenotypic features have been evaluated, and their functions have been investigated [5].

In inactive human breast tissue, c-Kit(+) telocytes have been identified in the non-epithelial region between tubuloalveolar structures and maintain a three-dimensional structure by forming multiple synapses with many immune cells, including macrophages, lymphocytes, and plasma cells [6,7]. It has been shown that the number and structural features of c-Kit(+) and CD34(+) telocytes in the rat mammary gland vary across different functional phases, such as resting, pregnancy, lactation, and involution, suggesting that telocytes might play a role in the tissue remodeling of the mammary gland and cell signaling [8].

Telocytes have also been detected in invasive breast carcinoma tumor stromata; however, their role in breast cancer remains poorly understood. One study found that the hetero-cellular connection of telocytes with other stromal cells is reduced and disrupted in the breast cancer microenvironment. In another study, c-Kit(+), CD34(+), and vimentin(+) telocytes promoted the growth of EMT6 breast cancer cells and inhibited apoptosis in vitro [9]. Babadag et al. evaluated the role of telocytes in the metastatic potential of breast cancer stem cells and showed that telocytes might reduce cancer metastasis [10]. These studies have demonstrated that telocytes, with their hetero-cellular connection with other breast stromal cells, are an essential component of normal mammary glands and tumor microenvironments; however, there are conflicting results for their functions regarding tumor behavior.

Non-cancerous components of the tumor microenvironment have emerged as a prominent focus. The tumor microenvironment plays an important role in tumor proliferation, propagation, and response to therapies. The tumor microenvironment in breast cancer exhibits significant differences between its molecular subgroups. Thus, defining specific cell populations within the tumor and microenvironment in these subgroups is essential to understanding tumor biology, potential therapeutic options, and resistance.

In this study, we investigated the expression of telocytes in different molecular subtypes of breast cancers and characterized their phenotypic characteristics and marker expression profiles.

## 2. Materials and Methods

This retrospective, cross-sectional study was approved by the Adnan Menderes University Non-Interventional Clinical Studies Ethics Committee (Approval No. 2021-56). Patients with breast cancer followed up at Adnan Menderes University, Faculty of Medicine, Department of Medical Oncology, were retrospectively screened, and patients with appropriate pathological blocks were included. Archival formalin-fixed, paraffin-embedded (FFPE) breast cancer tissue samples were used for analysis. Inclusion criteria comprised female patients aged 18 to 75 years with a histopathologically confirmed diagnosis of invasive breast cancer. Relevant demographic information was collected and analyzed to characterize the study population. Although there are four breast cancer molecular subgroups, we categorized Luminal A and B patients as a single group of hormone-receptor-positive patients. We had three breast cancer subgroups: the Luminal group: ER/PR (+) and HER2(–) patients; the HER-2(+) group: ER/PR(+/–) and HER2(+) patients; and the triple-negative group: ER/PR(–) and HER2(–) patients.

All laboratory procedures and analyses were conducted at the Department of Histology and Embryology, Faculty of Medicine, Gaziosmanpaşa University.

### 2.1. Immunohistochemistry

Immunohistochemical evaluation was performed to reveal the immunophenotype characteristics of and numerical differences between telocytes in the invasive breast cancer tissue of hormone receptor(+)/ERBB2(–), hormone receptor(+)/ERBB2(–), and hormone receptor(–)/ERBB2(–) patients. Tissue sections were deparaffinized and subsequently rehydrated by immersing sections in a graded alcohol series. Antigen retrieval was carried out using citrate buffer solution (Ph = 6) in a water bath at 95 °C for 30 min, and 0.3% hydrogen peroxide was applied for 20 min to inhibit endogenous peroxidase activity. To block non-specific binding sites, sections were incubated using block solution for 60 min at room temperature. The sections were subsequently treated with vimentin (Invitrogen (MA5-14564, Waltham, MA, USA) (1:200 dilution)), c-Kit (Invitrogen (34-8800, MA, USA) (1:100 dilution)), CD34 (Invitrogen (PA5-32322, MA, USA) (2:100 dilution)), and CD10 (Abcam (ab7349, Cambridge, UK) (2:100 dilution)) primary antibodies overnight at 4 °C, followed by rinsing with PBS. The sections were incubated with goat anti-rabbit HRP conjugate secondary antibody (Mouse- and Rabbit-Specific HRP/DAB IHC Detection Kit-Micro polymer (Abcam (ab236466, Cambridge, UK)) for 45 min at room temperature. DAB (3,3′-Diaminobenzidine) was applied for 5 min. Sections were then counterstained with Mayer’s hematoxylin for 2 min, dehydrated, cleared in xylene, and mounted with Entellan. Sections were photographed using a Nikon Eclipse E2000 light microscope attached to a Nikon DS-Fi1 camera (Nikon Corporation, Tokyo, Japan). The number of immunolabeled telocytes was determined manually in 10 fields per section in breast cancer tissue from each subgroup for quantitative evaluation. Specifically, telocytes were distinguished from other stromal cells (e.g., fibroblasts) based on their small cell bodies, moniliforms, and long telopodes despite overlapping immunophenotypes. To minimize inter-observer variability, all telocyte counts were performed by a single trained observer who was blinded to the molecular subtype of the samples.

Double immunolabeling with estrogen receptor (1/500) and c-Kit (1/200 dilution) antibodies was performed using Double Stain IHC M&R on human tissue (HRP/Green &AP/Red (Abcam) (ab210061, Cambridge, UK)). Briefly, for antigen retrieval, deparaffinized and rehydrated sections were treated with citrate buffer (pH 6.0) at 95 °C for 30 min. Endogenous peroxidase activity was blocked using 0.3% hydrogen peroxide for 20 min, followed by incubation with a blocking buffer for 45 min at room temperature to prevent non-specific binding. The sections were subsequently subjected to a mixture of primary antibodies ((c-Kit (Invitrogen (34-8800, MA, USA)) (1:100 dilution)) + Estrogen Receptor (Invitrogen MA5-15268, MA, USA)) overnight at 4 °C. The next day, sections were treated with a mixture of secondary antibody rabbit AP polymer and mouse HRP polymer at a 1:1 ratio, followed by chromogenic detection using permanent red and emerald chromogens. Counterstaining was performed using Mayer’s hematoxylin. Finally, sections were dehydrated in a graded alcohol series, and clearing was performed with xylene prior to mounting with organic mounting medium.

### 2.2. Statistical Analysis

Quantitative differences in telocytes were evaluated between molecular subtypes (the Luminal, HER2(+), and TNBC groups) and between the ER(+) and ER(–) patient groups, the PR(+) and PR(–) patient groups, the HER2(+) and HER2(–) patient groups, and the HER2(+)/hormone receptor(–) and HER2(+)/hormone receptor(+) patient groups. Additionally, we assessed correlations between tumor diameter and the number of telocytes and between the number of metastatic lymph nodes and the number of telocytes. Statistical analyses were performed using SPSS Statistics 22 (IBM-ABD). Data were tested for normality using the Shapiro–Wilk test. Multiple comparisons between means were performed using one-way ANOVA, and Tukey’s test was used as a post hoc test. The t-test was used to compare pairs of means. Data are provided as mean ± SE. *p* < 0.05 was considered statistically significant.

## 3. Results

### 3.1. Characteristics of Patients

A total of 58 cases of breast carcinoma were included in the study, with the following clinical information: 20 patients from the Luminal group, 19 from the HER2(+) group, and 19 from the TNBC group. All patients were followed up at the Adnan Menderes University Application and Research Hospital Oncology Clinic. In total, 17.2% of the patients were under the age of 40, and 82.8% were over the age of 40. Their age range was 21–74 years, with a mean age of 54.87 ± 1.794 years. Tumor diameter was <2 in 35.1% of the patients, between 2 and 5 cm in 50.9%, and >5 in 14% of the patients. Patients were diagnosed with 79.3% invasive ductal carcinoma, 8.6% with invasive lobular carcinoma, 3.4% with medullary carcinoma, 3.4% with mixed breast carcinoma (invasive ductal carcinoma and invasive lobular carcinoma), 1.7% with solid papillary carcinoma, and 3.4% with metaplastic carcinoma. The percentage of patients with 0 metastatic lymph nodes was 51.8%, the percentage of patients with 1–3 lymph nodes was 19.6%, the percentage of patients with 4–9 lymph nodes was 21.4%, and the percentage of patients with 10 or more was 7.1%. The percentage of ER(–) patients was 52.6%, the percentage of ER(+) patients was 47.4%, the percentage of PR(–) patients was 54.4%, and the percentage of PR(+) patients was 45.6%. The percentage of HER2(–) patients was 67.2%, and the percentage of HER2(+) patients was 32.8% (Table 1).

### 3.2. Relationship Between Telocyte Number and Molecular Subtype of Invasive Breast Cancer

**Figure 1:**
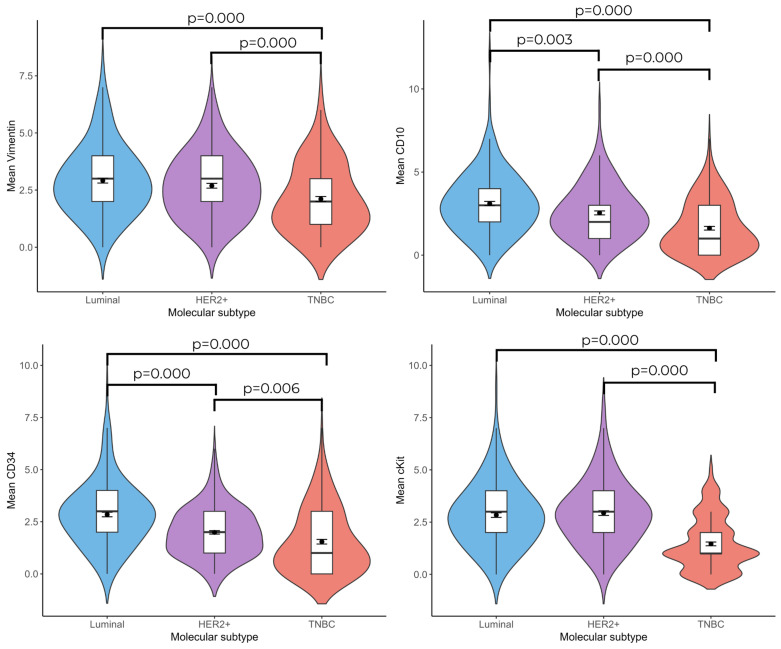
Violin plots show the relationship between vimentin(+), CD10(+), CD34(+), and c-Kit(+) telocyte numbers and molecular subtypes of breast cancer. (n = 20 for Luminal group, n = 19 for HER2(+) group, and n = 19 for TNBC group).

**Figure 2:**
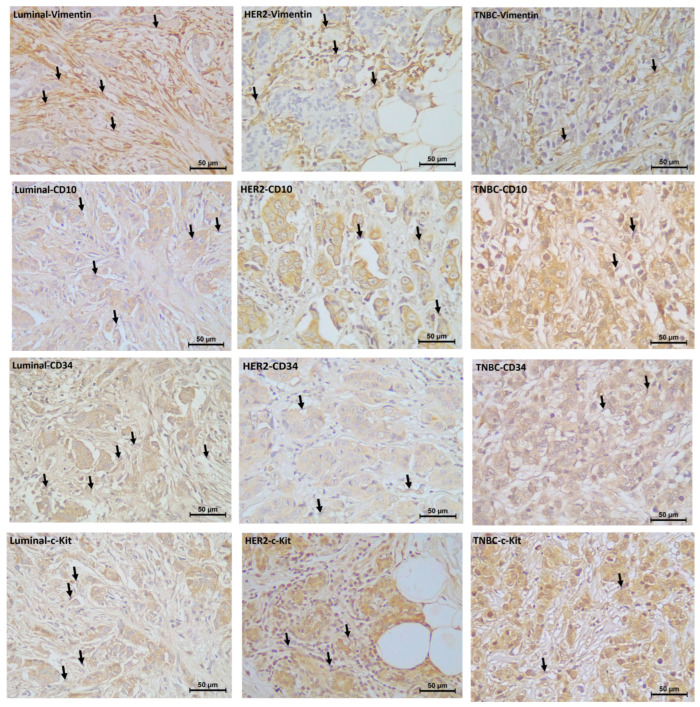
Immunohistochemical labeling of vimentin(+), CD10(+), CD34(+), and c-Kit(+) telocytes in molecular subtypes of breast cancer. Arrows indicate immunoreactivity for telocytes in tissue (40×; scale-bar = 50 µm for all images).

According to the immunohistochemical labeling results, the number of vimentin(+), CD10(+), CD34(+), and c-Kit(+) telocyte cells was found to be statistically significantly higher in the Luminal and HER2(+) groups compared with the TNBC group ((Luminal vs. TNBC; vimentin: 2.92 ± 0.107–2.11 ± 0.110; *p* = 0.000, CD10: 3.11 ± 0.128–1.62 ± 0.112; *p* = 0.000, CD34: 2.85± 0.110–1.54 ± 0.110; *p* = 0.000, and c-Kit: 2.84 ± 0.110–1.46 ± 0.09; *p* = 0.000), (HER2(+) vs. TNBC; vimentin: 2.69 ± 0.104–2.11 ± 0.110; *p* = 0.000, CD10: 2.54 ± 0.117–1.62 ± 0.112; *p* = 0.000, c-Kit: 2.93 ± 0.110; *p* = 0.000, CD34: 1.99 ± 0.083–1.54 ± 0.110; *p* = 0.006)). No significant difference was observed between the Luminal and HER2(+) breast cancer groups in terms of vimentin(+) and c-Kit(+) telocyte cell numbers; the number of CD10(+) (3.11 ± 0.128–2.54 ± 0.117; *p* = 0.003) and CD34(+) (2.85 ± 0.110–1.99 ± 0.083; *p* = 0.001) telocyte cells were significantly higher in the Luminal group than in the HER2(+) group (Figure 1 and Figure 2).

### 3.3. Relationship Between Telocyte Number and ER, PR, and HER2

**Figure 3:**
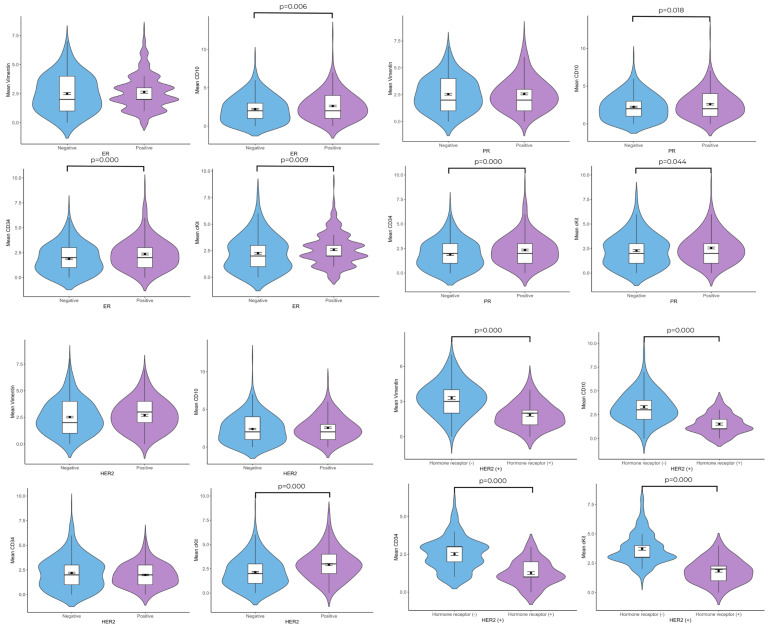
Violin plots show quantitative differences in telocytes between ER(+) and ER(–) patient groups; PR(+) and PR(–) patient groups; HER2(+) and HER2(–) patient groups; and HER2(+)/hormone receptor(–) and HER2(+)/hormone receptor(+) patient groups (n = 30 for ER(+) and n = 27 for (ER(–); n = 31 for PR(+) and n = 26 for PR(–); n = 39 for HER2(+) and n = 19 for HER2(–); n = 11 for HER2(+)/hormone receptor(–) and n = 8 for HER2(+)/hormone receptor(+)).

**Figure 4:**
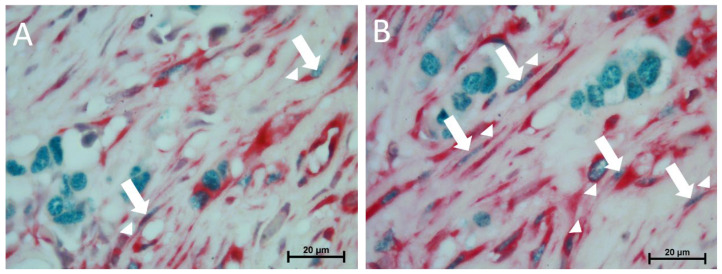
Double immunolabeling for c-Kit (red) and ER (green) (**A**) HER2(+)/hormone receptor(+) group; (**B**) Luminal group telocytes are identifiable as c-Kit(+)/ER(+) stromal cells with characteristic extensions, 100×. Arrows: nuclei of telocytes (green); arrowheads: extensions of telocytes (red).

When ER(+) patients and ER(–) patients were compared in terms of telocyte cell number, the CD10(+) (2.63 ± 0.090–2.22 ± 0.098; *p* = 0.006), CD34(+) 2.36 ± 0.095–1.88 ± 0.082; *p* = 0.000), and c-Kit(+) (2.60 ± 0.093–2.25 ± 0.094; *p* = 0.004) telocyte cell numbers were found to be higher in the ER(+) group. Although the ER(+) group contained a higher vimentin(+) telocyte number, the difference was not statistically significant. According to the c-Kit/ER double immunolabeling results, these cells were also ER(+). In the PR(+) group, higher numbers of CD10(+) (2.60 ± 0.111–2.25 ± 0.098; *p* = 0.018), CD34(+) (2.35 ± 0.96–1.88 ± 0.82; *p* = 0.000), and c-Kit(+) (2.56 ± 0.095–2.29 ± 0.094; *p* = 0.044) telocytes were observed in the connective tissue than in the PR(–) group. The vimentin, CD10, and CD34 immunolabeling sections revealed that there were no significant differences in the mean number of telocytes in the HER2(+) group compared with the HER2(–) group. The c-Kit(+) (2.93 ± 0.110–2.15 ± 0.079; *p* = 0.000) telocyte cell number was found to be higher in the HER2(+) group. When HER2(+)/hormone receptor(–) patients and HER2(+)/hormone receptor(+) patients were compared in terms of telocyte cell number, the vimentin(+) (1.87 ± 0.119–3.27 ± 0.131; *p* = 0.000), CD10(+) (1.52 ± 1.024–3.27 ± 0.131; *p* = 0.000), CD34(+) (1.25 ± 0.091–2.51 ± 0.101; *p* = 0.000), and c-Kit(+) (1.85 ± 0.111–3.70 ± 0.127; *p* = 0.000) telocyte cell numbers were found to be higher in the HER2(+)/hormone receptor(–) group (Figure 3 and Figure 4).

### 3.4. Relationship Between Telocyte Number and Metastatic Lymph Node Number

**Figure 5:**
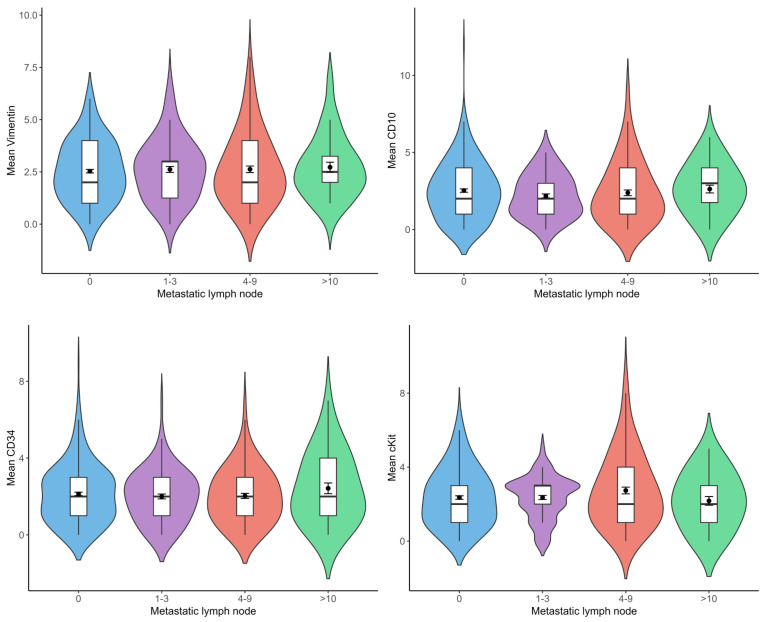
Violin plots show the relationship between vimentin(+), CD10(+), CD34(+), and c-Kit(+) telocyte numbers and number of metastatic lymph nodes (n = 29 for 0, n = 11 for 1–3, n = 12 for 4–9, n = 4 for >10).

There was no statistically significant difference between the number of metastatic lymph nodes and vimentin(+), CD10(+), CD34(+), and c-Kit(+) telocyte cell numbers (Figure 5).

### 3.5. Relationship Between Telocyte Cell Number and Tumor Diameter

**Figure 6:**
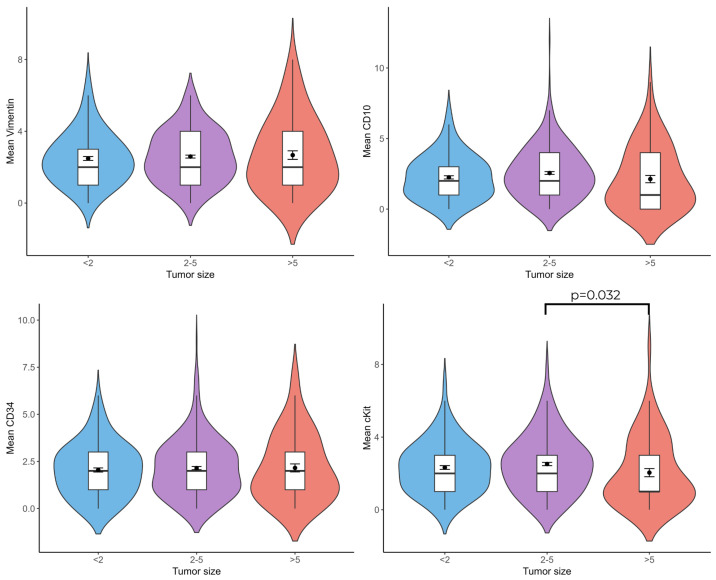
Violin plots showthe relationship between vimentin(+), CD10(+), CD34(+), and c-Kit(+) telocyte numbers and tumor size. (n = 20 for <2, n = 29 for 2–5, n = 8 for >5).

When tumor diameter and vimentin(+), CD10(+), CD34(+), and c-Kit(+) telocyte cell numbers were compared, c-Kit(+) (*p* = 0.032) telocytes were less numerous in patients with a tumor diameter larger than 5 cm than in patients with a tumor diameter of 2–5 cm (Figure 6).

## 4. Discussion

The tumor microenvironment includes tumor cells, local factors, immune cells, endothelial cells, and stromal cells. The interaction between tumor cells and their microenvironment is crucial in tumor proliferation, progression, and treatment response [11]. Telocytes are stromal cells that have a particular cell body and very thin and long cytoplasmic extensions (telepodes) and are considered to play a crucial role in the tumor microenvironment. These cells construct a network through telepodes and contact with parenchyma, immune, and cancer cells [12]. Breast cancer is a highly heterogeneous complex of diseases, with a spectrum of many subtypes with varying distinct histological and biological features that influence clinical outcomes. Distinct molecular subtypes of breast cancer exhibit unique tumor microenvironment profiles [13]. In this study, we evaluated the quantitative and immunohistochemical aspects of telocytes in human breast cancer tissue and compared them according to molecular subtypes.

Telocytes are found in various organs and tissues and may exhibit different morphological and phenotypical features. Telocytes may also express aberrant stem cell markers in response to widespread gene mutations in the tumor microenvironment. Therefore, there is no single universal marker for identifying telocytes at present. While some studies have reported c-Kit(+) cells as telocytes in breast tissue, others have defined telocytes with the CD34(+)/CD10(±)/c-Kit(–) immunophenotype [6,7]. In our study, telocytes were marked with vimentin, CD10, CD34, and c-Kit antibodies, and quantitative evaluation was performed considering their structural features.

In this study, telocyte numbers differed in terms of immunological characteristics according to breast cancer subtype. For example, Luminal and HER2(+) breast cancers exhibited higher numbers of CD34(+)/CD10(+)/c-Kit(+)/vimentin(+) telocytes than triple-negative breast cancers. Additionally, Luminal cancers had higher numbers of CD10(+) and CD34(+) telocytes than the HER2(+) subgroup. The variable telocyte numbers across breast cancer subtypes could be a reflection of the tumor microenvironment’s heterogeneity; for example, Luminal tumors have a relatively organized stromal architecture, while TNBC tumors exhibit more fibrotic and immunosuppressive stroma [14]. As connective cells that provide mechanical support, telocytes significantly contribute to forming a supportive framework via homo- and hetero-connections within the mammary gland. Detecting telocytes with different markers in different molecular subtypes shows that these cells are related to the microenvironment and may have different functions in response to environmental factors. However, the specific role of telocytes in regulating tumor behavior has yet to be elucidated. While telocytes have been hypothesized to participate in angiogenesis, malignant cell migration, invasion, and proliferation, further assessment is required regarding their functional roles.

In our study, telocytes in ER(+) invasive breast cancer tissues were shown to be c-kit/ER(+) cells using double immunohistochemical labeling. This dual positivity is consistent with previous hypotheses that telocytes may behave as hormonal sensors, particularly in tissues where estrogen signaling plays a regulatory role [15]. However, further functional studies are essential to determining whether these c-Kit(+)/ER(+) telocytes are actively involved in tumor progression or represent cells affected by the hormonal microenvironment.

c-Kit(+) telocytes markedly decreased in tumors over 5 cm in diameter compared with those measuring 2–5 cm. This finding suggests the possible role of c-Kit(+) telocytes in regulating tumor size. Telocytes can show different expression patterns that could be related to distinct functions. c-Kit(+) cells have been shown to regulate immune responses, maintain tissue homeostasis, and express proinflammatory cytokines to modulate immune responses and suppress tumor growth via paracrine signaling [16,17]. A decrease in c-Kit(+) telocytes is linked to more aggressive tumors.

This study has some limitations. Since it was retrospective, we used paraffin blocks, and ultrastructural evaluation could not be performed. Although various markers (CD34, CD10, c-Kit, and vimentin) were used to define telocytes, the specificity of these markers remains controversial, as many are also expressed by other stromal or immune cells. The relatively small sample size may limit the general applicability of our findings across broader breast cancer populations. Future studies should consider functional validation through in vitro co-culture experiments to investigate telocyte–tumor interactions, as well as spatial transcriptomics to better define the role of telocytes within the tumor microenvironment.

## 5. Conclusions

The current study demonstrated quantitative differences in telocyte marker expression—specifically vimentin, CD10, CD34, and c-Kit—across molecular subtypes of invasive breast cancer. Additionally, the presence of ER-immunopositivetelocytes was identified within the breast cancer microenvironment. These findings contribute to a descriptive understanding of the telocyte distribution in breast cancer and highlight the need for further functional studies to clarify their potential roles in tumor biology.

## Figures and Tables

**Table 1:** Clinical features of patients.

Sociodemographic/Clinical Features	Classification	Number	Percentage (%)
Age (year)	<40 >40	10 48	17.2 82.8
Tumor size (cm)	<2 2–5 >5	20 29 8	35.1 50.9 14.0
Molecular subtype	Luminal HER2(+) TNBC	20 19 19	34.5 32.8 32.8
Histopathological subtype	Ductal Lobular Others	46 5 7	79.3 8.6 11.9
Number of metastatic LNs	No metastasis 1–3 4–9 ≥10	29 11 12 4	51.8 19.6 21.4 7.1
ER	Negative Positive	30 27	52.6 47.4
PR	Negative Positive	31 26	54.4 45.6
HER2	Negative Positive	39 19	67.2 32.8

## Data Availability

All immunobiological micrographs and graphical results are original data. The immunobiological images and raw datasets analyzed in the current study are available upon request from Özlem Yersal (yersal1978@yahoo.com).

## Abbreviations

The following abbreviations are used in this manuscript:

ER	Estrogen Receptor
PR	Progesterone Receptor
HER2	Human Epidermal Growth Factor 2
TNBC	Triple-Negative Breast Cancer
